# Survey of knowledge for diagnosing and managing prediabetes in Latin-America: cross-sectional study

**DOI:** 10.1186/s13098-019-0500-4

**Published:** 2019-12-04

**Authors:** Jennifer Garay, Paul A. Camacho, Jose Lopez-Lopez, Juliana Alvernia, Marcela Garcia, Daniel D. Cohen, Carlos Calderon, Patricio Lopez-Jaramillo

**Affiliations:** 1 Research Department, Fundacion Oftalmologica de Santander (FOSCAL) Internacional, Calle 158 # 20-95, Consultorio 101/102, Floridablanca, Santander Colombia; 2 Medical School, Universidad Autónoma de Bucaramanga (UNAB), Floridablanca, Colombia; 3 Instituto Masira, Medical School, Universidad de Santander (UDES), Bucaramanga, Colombia; 4 Eugenio Espejo Medical School, Universidad UTE, Quito, Ecuador; 5 Fundación Santandereana de Diabetes (FUSANDE), Cra. 33 #46-45, Bucaramanga, Santander Colombia

**Keywords:** Latin America, Prediabetes, Knowledge, Diagnosis, Management

## Abstract

**Background:**

Prediabetes has been proposed as a risk factor for the development of type 2 diabetes mellitus (DM2) and cardiovascular disease (CVD). Despite the clinical importance of prediabetes, little is known about the level of knowledge, beliefs and barriers to screening and treating prediabetes amongst care health providers in Latin America. The aim of the present survey was to evaluate the knowledge and beliefs about prediabetes amongst in Latin American health care providers.

**Methodology:**

In a cross-sectional study, we adapted the written survey designed by the Johns Hopkins University group, and applied it to health care providers across Latin America during three meetings, in 2017, and with physicians from primary care centers in Bucaramanga, Colombia convened in 2017. The survey consisted of questions under four headings, diabetes screening, management of prediabetes, pharmacological treatment—metformin use, and demographic information. We perform a descriptive analysis to determine the differences in responses between different medical specialties.

**Results:**

The majority of the care providers that answered the survey were Colombian physicians, 54.5% of respondents had 10 years or more since completing their training and more women responded. Only 9.5% identified the 12 prediabetes risk factors described in the literature. The most common risk factor identified was a family history of diabetes, followed by overweight, a sedentary lifestyle and dyslipidemia, while ethnicity was the risk factor least commonly. 47.1% answered that laboratory tests to detect prediabetes are fasting glucose and HbA1C, 82.5% correctly identified fasting plasma glucose as the best test, 35.9% correctly responded that to the recommended weight loss goal is 5 to 7% and 49.1% that 150 min is considered the minimum level of physical activity per week. 78% agreed that the identification and treatment of prediabetes is important. 56% believed that patients with prediabetes progress more rapidly to diabetes and 40.6% considered that metformin could reduce the risk of diabetes in patients already diagnosed with prediabetes.

**Conclusion:**

These results demonstrate that there are important gaps in the knowledge of the diagnosis, clinical implications and management of prediabetes amongst Latin America health providers.

## Background

Prediabetes, as defined by the American Diabetes Association guidelines, is the term used for those individuals who’s glucose levels do not meet the criteria for diabetes but are sufficiently elevated to increase the risk of cardiovascular disease (CVD) [1]. Although it is not a distinct clinical entity, prediabetes increases the risk of developing diabetes mellitus 2 (DM2) 3–10 times and is associated with a progress towards DM2 of 10% annually [2, 3]. Prediabetes includes impaired fasting glucose (IFG) and/or impaired glucose tolerance (IGT) [4]. In 2015 it was estimated that 84.1 million people had prediabetes, but only 11.6% of these people had been informed of this by a health provider [5, 6]. The International Diabetes Federation (IDF) estimates that globally there are 280 million people with IGT [6]. The prevalence of prediabetes in Latin America is reported to be 9.8% [7] and in this population, prediabetes was shown to be the most frequent glucose disorder in patients with acute myocardial infarction (AMI) [8]. In the United States of America (USA), despite the clinical importance of prediabetes, knowledge of the condition and its detection and treatment is very low amongst primary health care providers [7], and it is relatively unknown in Latin America. Therefore, the objective of this survey was to assess the state of knowledge and beliefs about prediabetes among health care providers in Latin America and differentiate between the specialties.

## Methods

This is a cross-sectional study in which a written survey designed by the Johns Hopkins University group [9] was adapted and translated it into Spanish. The survey uses the American Diabetes Association (ADA) criteria for the diagnosis of prediabetes and includes questions about the management, practices, attitudes, and beliefs in prediabetes [10]. The survey comprises four components. Questions in the first section are about diabetes screening, including knowledge about risk factors, methods, and guidelines for screening. The second section asks about the management of prediabetes, including initial therapy, drug therapy, and follow-up. The third contains specific questions about pharmacological treatment, including the prescription of metformin and barriers about its use. The final part includes demographic information, type of medical specialty, training time, and consultation time per week. The survey was distributed and completed by health care providers during the X Latino-American Internal Medicine meeting/XXVI Colombian Internal Medicine Association/American College Physicians meeting, Cartagena, and the 84th diabetes update Course from the Colombian Diabetes Federation, Barranquilla, and the Third Central American Diabetic Foot Congress at Tegucigalpa, Honduras, all occurring during August 2017. At the same time, the survey was also delivered to physicians in the health centers of the Institute of Health of Bucaramanga, Colombia (ISABU). Survey data was entered into a web portal. As local regulations state that this kind of observational studies do not require ethical clearance, the survey was not submitted to an ethical committee.

### Statistical analysis

We perform a descriptive analysis. Qualitative variables were summarized in absolute and relative frequencies. The quantitative variables were summarized with measures of central tendency, position and dispersion according to the frequency distribution. The difference in the results of the study were estimated using the Chi-square test and the exact Fischer test. For questions using a Likert scale, we dichotomized the answers to agree; by combining agree and, strongly agree and disagree; by combining neutral, disagree and strongly disagree. All statistical analysis was carried out using statistical software Stata, version 11.0 (Stata Corporation, College Station, TX, USA). The level of significance of the study is 5%.

## Results

The majority of the care providers that responded to the survey were physicians (94.3%), and 5.7% were nurses, nutritionist and physical therapists (Table 1). 42.1% had 10 years or more since completing their training. There were more females (53.7%) than males. Table 1 summarizes the characteristics of survey respondents.

**Table 1:** Characteristics of survey respondents (n = 373)

Provider characteristics	N (%)
Specialty	
General medicine	189 (60.2)
Specialty medicine	107 (34.1)
Internal medicine	87
Family medicine	11
Other specialties	9
Others	18 (5.7)
Provider type	
Physicians	296 (94.3)
Nurse	12 (3.8)
Nutritionist	5 (1.6)
Physical conditioner	1 (0.3)
Number of years since completing training^a^	
< 5 years	112 (42.4)
5–10 years	41 (15.5)
10+ years	111 (42.1)
Gender	
Female	174 (53.7)
Male	150 (46.3)

10 Missing

^a^40 Missing

### Knowledge of risk factors and laboratory criteria, prediabetes practice and management

Table 2 summaries knowledge of diagnoses criteria and management of prediabetes. The principle approaches for initial management of prediabetes used by the providers were: counseling on diet changes and physical activity to lose weight (94.9%), and referral of the patient to a nutritionist (57.3%). Seventy percent considered that they should repeat laboratory tests 3 months after the diagnosis of prediabetes with further follow up 3 months after that (75.1). Almost 80% of the physicians that had patients with prediabetes (without progression to diabetes) had prescribed less than 25% with metformin. Table 3 shows self-reported practice in patients with prediabetes.

**Table 2:** Knowledge for diagnoses criteria and management of prediabetes

	N (%)	P value
Correct identification of diabetes laboratory criteria; fasting glucose	275 (83.3)	0.001
Correct identification of diabetes laboratory criteria; HbA1c	192 (58.2)	0.055
Correct identification of prediabetes laboratory criteria; fasting glucose	168 (48.2)	0.349
Correct identification of prediabetes laboratory criteria; HbA1c	49 (15.1)	0.481
Correct body weight loss recommendation; 5–7%	107 (35.1)	0.172
Correct physical activity recommendation; 150 min/week	167 (51.7)	0.391
Correct initial management recommendation; referral to behavioral weight loss program	7 (3.03)	0.519

**Table 3:** Self-reported practice in prediabetes patients

Practice	N (%) current study	P value
Initial management approach		
Counseling on diet changes and physical activity	313 (94.9)	0.063
Refer to nutritionist	189 (57.3)	0.002
Refer to behavioral weight loss program	98 (29.7)	0.132
Discuss starting metformin	102 (30.9)	0.832
Refer for bariatric surgery	10 (3.0)	0.934
Repeat laboratory tests		
3 months	233 (70.8)	< 0.001
6 months	62 (18.8)	0.001
1 year	23 (7.0)	0.754
2 years	2 (0.6)	–
No specific recommendation	9 (2.8)	0.900
Return for follow-up clinic visit		
3 months	247 (75.1)	< 0.001
6 months	45 (13.7)	0.004
1 year	16 (4.9)	0.992
2 years	1 (0.3)	–
No specific recommendation	1 (0.3)	–
% Patients with prediabetes prescribed metformin		
0%	72 (21.9)	0.766
1–5%	93 (28.3)	0.343
> 5–25%	76 (23.1)	0.746
> 25–50%	41 (12.5)	0.586
> 50–75%	29 (8.8)	0.784
> 75%	18 (5.5)	0.809

### Attitudes, beliefs and barriers

There is strong agreement (78%) that the identification and management of prediabetes is important, but just 46% strongly agreed that this would help identify the means to treat comorbidities like hypertension, and only 56% believed that patients with prediabetes progress more rapidly to diabetes. Only 40.6% considered that metformin could reduce the risk of diabetes in patients that have already been diagnosed with prediabetes.

Amongst the barriers that the providers identified to be effective in lifestyle changes, (88.4%) agreed and strongly agreed that there was a lack of patient motivation, (72.5%) thought that the patients did not consider lifestyle changes important. 45% considered that weight-loss and physical activity goals were not achieved due to lack of resources, or to financial limitations. Table 4 summarizes the barriers for interventions in prediabetes patients.

**Table 4:** Barriers for interventions in prediabetes patients

	N (%)	P value
Barriers to lifestyle modification (strongly agree and agree)		
Patient’s lack of motivation	290 (88.4)	0.488
Patient’s physical limitation in doing activity	200 (61.7)	0.040
Lack of weight loss resources for patient	148 (45.3)	0.003
Lack nutrition resources for patient	176 (53.7)	0.006
Patients do not think it is important to make these changes	235 (72.5)	0.404
Financial limitations	161 (49.2)	0.346
Barriers to metformin use (strongly agree and agree)		
Patients dislike taking medications	228 (70.8)	0.001
Medication cost to patient	108 (33.4)	0.188
Poor patient adherence	246 (76.2)	0.232
Potential side effects	188 (58.6)	0.001
Providers’ lack of awareness of clinical guidelines for metformin use	197 (61.4)	0.270
Lack of FDA approval for metformin use in prediabetes	97 (30.8)	0.878
Interventions to improve management of prediabetes (strongly agree and agree)		
More time for doctors to counsel patients	275 (83.8)	0.768
More educational resources for patients	291 (88.7)	0.816
Improved access to diabetes preventive programs	302 (92.4)	0.199
Improved nutrition resources for patients	278 (84.8)	0.006
Improved access to weight loss programs	282 (86.5)	0.229
Improved access to bariatric surgery	103 (31.6)	0.613

Amongst the interventions that could be improved in the management and treatment of prediabetes, (92.4%) believed that there is a need to improve the access to prevention programs and 58% believed that there is a need for more, and better, educational and nutritional resources. The reasons providers gave for prescribing metformin in patients with prediabetes were: risk of diabetes and obesity (73%), HbA1c > 6% (53%), lack of response to lifestyle intervention (52%) and family history of diabetes (47%). Providers agreed and strongly agreed that the main barriers to the use of metformin is poor patient adherence (76.2%) and because patients did not like to take medicines (70.8%). Moreover, the lack of awareness about the recommendations of the clinical guidelines for metformin use in prediabetes is high (61.4%).

### Knowledge by type of speciality of the provider

**Fig. 1:**
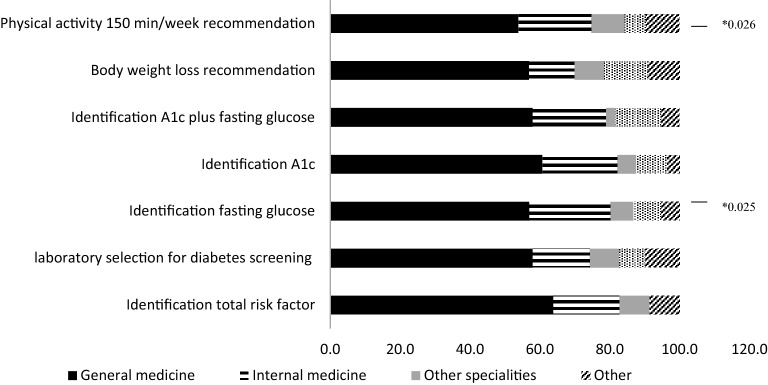
Correct identification of the diagnosis criteria and recommendations for prediabetes: a comparison between health providers

There was no difference in the level of knowledge about the risk factors that might prompt screening for prediabetes and diabetes between doctors in general medicine, internal medicine, other specialties and other health providers. Similarly, there were no differences in the laboratory criteria for diagnosing prediabetes with the use of HbA1c, in the minimum weight loss recommendation or the selection of laboratory tests for screening. However, there were differences in the correct identification of recommended minimum physical activity and in the fasting glucose values that diagnosed prediabetes (Fig. 1).

## Discussion

The present study shows that there is insufficient knowledge about the diagnosis, clinical implications and management of prediabetes amongst the Latin American health providers. The participants answered correctly that a family history of diabetes in a first-degree relative is a main risk factor and the main criteria to screen their patients. However, few recognized ethnicity as a risk factor or that prediabetes is a risk factor for CVD. Despite 75% of those surveyed agreeing that lifestyle modification can reduce the risk of diabetes, 50% correctly identified guidelines recommendations for minimum physical activity and target weight-loss [1]. There was a strong perception that low adherence of patients to lifestyle modifications is due to the lack of motivation and a perception that these changes have clinical impact. However, there is also a lack of familiarity with weight loss programs and skepticism about the effectiveness of these programs among health care providers [11–13]. There is an important substantial underprescription of metformin in the treatment of prediabetes, despite the published Latin American and Colombian Consensus recommending its use if the goal of glycemia is not achieved after 3 months of lifestyle changes [1, 3, 14]. Additionally, the ADA guidelines [1] recommend the use of metformin for the prevention of development of DM2 in subjects with prediabetes, especially in those with body mass index > 35 kg/m^2^, over 60 years old, and in women with a history of gestational diabetes (recommendation grade A). This recommendation is based mainly on the results of the Diabetes Prevention Program [15, 16], which showed the importance of using metformin in high-risk subjects. Of the 3234 subjects with IFG and body mass index (BMI) > 24 included in the study, 1079 were randomized to intensive lifestyle intervention, 924 to metformin treatment, and 932 to placebo. At 2.8 years of follow-up, lifestyle changes were the most effective intervention for the reduction in the incidence of DM2 (58% compared to placebo). However, metformin was effective in reducing this incidence by 31% compared to the placebo. Moreover, at the 15-year follow-up, the incidence of diabetes was reduced by 18% in the metformin group (0.82, 0.72–0.93; p = 0.001) compared to placebo [17]. Lifestyle changes are the first line and the cornerstone of dysglycemia management. However, given the particular context of our region regarding social and economic, where there are limited time and resources to implement adequate monitoring programs, the addition of the pharmacological strategy as a compliment in the management could be a correct intervention. In addition, the Diabetes Prevention Program of India (IDPP) that resemblances our socioeconomic context showed that changes in lifestyle and metformin reduced the progression to DM2 in a similar proportion, 28.5% (95% CI 20.5 to 37.3%) vs 26.4% (95% CI 19.1 to 35.1%), respectively [18]. The results of our study are worrisome since we have previously shown that 49% of patients with a first AMI were unaware that they had prediabetes, which is not only associated with a higher risk of AMI, but also to lower survival rates following it [8]. Moreover, there is evidence that the benefit of treating prediabetes is the reduction in the risk of progression to diabetes and coronary atherosclerosis [18–20].

The general medicine physicians, who in Latin America are the first line or gatekeeper of primary care provision [21, 22], had the best survey performance in comparison with the internal medicine and other specialists. This is an unexpected result, which may be related to the fact that many of the general medicine physician’s surveyed work in direct government preventive care programs. Nonetheless, the overall knowledge of detection and management of prediabetes can be considered too low as previously reported in health providers in a region of the USA [9]. The adoption of the guidelines proposed by the World Health Organization (WHO), including the “25 × 25” strategy, can improve the detection and control of the main cardiovascular risk factors [23]. For example, the Heart Outcomes Prevention Evaluation 4 (HOPE-4) [24], community-based implementation study showed that task-sharing with non-physician health workers for the education of patients, the supply of free medicines, and the participation of family and friends led to a more than 40% reduction in estimated cardiovascular risk at 10 years and doubled the control of hypertension in comparison to the control group (usual medical care). This strategy could be adapted for the early recognition of prediabetes and its management at the community and primary care health providers level.

The present study has some limitations. The survey was not validated; however, due to the characteristics of the people evaluated, the questions were designed to evaluate concepts about universal definitions. Most of the participants surveyed were physicians, dissimilar to the percentage of nurses included in the survey that was low because of the low attendance rates of this group to the events. These events were predominantly directed to physicians. There was a lack of complementary questions such as around the knowledge of the glucose tolerance test. However, this is also a limitation of the original survey. The providers surveyed attended to three different medical meetings which included prediabetes related topics and were completed during the sessions, this may lead to a higher risk of information bias. We therefore need to survey a sample in a different context to determine the reproducibility of our results, particularly considering that our sample was not representative of Latin America as a whole, with as most participants were from Colombia.

## Conclusions

Our results demonstrate important gaps in the knowledge of the diagnosis, clinical implications and management of prediabetes amongst the Latin America health providers. These results are of concern since in recent decades there has been a substantial increase in the prediabetes burden in Latin America, associated with an increased risk of DM2 and CVD [3, 7]. Moreover, there is evidence that early identification and management of prediabetes may prevent or delay the progression to diabetes and cardiovascular events [18–20]. Our results suggest that there is an urgent need to widely implement and improve the teaching of prediabetes in medical and health schools and in continuing medical education programs.

## Data Availability

The author of correspondence has available the total data.

## Abbreviations

DM2: diabetes mellitus type 2
HbA1C: glycosylated hemoglobin
IFG: fasting impaired glucose
IGT: impaired glucose tolerance
CVD: cardiovascular disease
IDF: International Diabetes Federation
AMI: acute myocardial infarction
USA: United States of America
ADA: American Diabetes Association
ISABU: Institute of Health of Bucaramanga
